# Professor Brian Fox

**DOI:** 10.1038/sj.bjc.6690675

**Published:** 1999-09

**Authors:** D G Harnden

**Affiliations:** Paterson Institute for Cancer Research Christie Hospital NHS Trust, Wilmslow Road, Manchester, M20 9BX


*British Journal of Cancer* (1999) **81**(2), 189
© 1999 Cancer Research Campaign
Article no. bjoc.1999.0675

Obituary

### Professor Brian Fox

Brian Fox died suddenly on 28th March 1999 at the age of 69. He leaves behind a quite remarkable legacy of contributions to cancer research. Unusually, he also achieved international distinction in a second scientific field, the study of lichens, as well as having a myriad of other interests.

Brian was born in South Wales in 1929 and although he spent most of his life in the north of England he remained a proud Welshman. He graduated in Chemistry in 1950 from Durham University and continued there to obtain his PhD in 1954. His doctoral thesis was on the isolation and characterization of plant extracts such as the alkaloids of the lupin. This interest in natural history, combined with a love of chemistry, shaped the rest of his career. After national service for 2 years in the Royal Signals Regiment he took up a post in the Paterson Laboratories, at the renowned Christie Hospital in Manchester, where he remained for the rest of his career with the exception of a sabbatical year at Yale University as a Cancer Research Campaign (CRC) Fellow. He became head of the Department of Experimental Chemotherapy at the Paterson Laboratories and was appointed to a Personal Chair in the University of Manchester in 1980 and as Deputy Director of the Paterson Institute for Cancer Research in 1984. He retired in 1993.

Brian Fox's interest in chemotherapeutic agents arose out of his early work with Harold Jackson on agents which could modulate fertility. He was particularly interested in understanding the role of geometrical shape in the activity of drugs. This was particularly true of his studies on the interaction of allkane sulphonate drugs with chromatin. These studies led ultimately to the introduction of methylene dimethane sulphonate (MDMS) into clinical trials. In his later career he became particularly interested in developing drugs from natural sources. This research was facilitated by the close relationship he built up with Professor Bob Pettit of Arizona State University, and led to the introduction of a range of new agents, including bryostatin I, into clinical trials under the sponsorship of the CRC. He also realized the potential of Chinese Traditional Medicine as a source of new agents. With the help of Chinese colleagues a number of novel structures were isolated and are now being evaluated as potential anticancer agents.

Brian, together with Tom Connors, was the driving force behind the CRC Phase I/II Clinical Trials Committee and he served as operational secretary from 1980 to 1992. The underlying concept was a system that would fast-track potential cancer chemotherapeutic agents into clinical trials. The mechanism established by this group has been internationally recognized and many other countries have adopted similar systems. In the UK this Committee has evolved into a sophisticated collaboration between cancer research organizations and is now beginning to reap the benefits of the foresight of Brian Fox and Tom Connors. His international distinction was also recognized by his membership of the council of the European Organisation for Research and Treatment of Cancer (EORTC). He was Chairman of the EORTC Screening and Pharmacology Group and its New Drug Development Group, and also a member of the prestigious National Cancer Institute/ EORTC/CRC Joint Steering Committee.

For Brian, lichens were no trouble – he loved them. He was a very active member of the British Lichen Society and served as Vice President and President. Not only did the study of lichens take him to the hills of Derbyshire and other wild and remote regions which he greatly enjoyed, it also provided a basis for some valuable work in monitoring environmental pollution for local councils in the North of England. He served on the Advisory Committee for the British Lichen Flora. His botanical interests led to his election in 1960 as a Fellow of the Linnean Society, and in 1986 he became a Council Member. He was a familiar figure in the herbariun of the University of Manchester Museum and he was, at the time of his death, involved in preparing exhibits for the Wellcome Science for Life Exhibition, which is shortly to transfer to Manchester.

He was also the unofficial archivist for the Christie Hospital and had amassed a large collection of artefacts, documents and recorded conversations with many of the distinguished figures who contributed to the development of cancer treatment and cancer research. A booklet based on this material was published a few years ago and he had completed many chapters of a much larger work. This material remains a wonderful opportunity for further study on the history of cancer research and treatment.

He was an accomplished pianist and an artist with a special interest in portrait painting. Those who worked most closely to him know that he could be a demanding task master. However, his enthusiasm for all he did was infectious and a number of his students and research fellows have themselves become internationally known figures in the field of cancer chemotherapy. His scientific philosophy and integrity live on in the work of many students and colleagues who thrived under his guidance.

Sadly his wife, Margaret, also a distinguished scientist whom he met and worked with at the Paterson Institute, died quite suddenly several years ago. He will be greatly missed by his sister Mary, other relatives and his many friends and colleagues around the world.

Truly, this was a remarkable and busy life; a life's work well done.

*Professor DG Harnden*
*Director*
*Paterson Institute for Cancer Research*
*Christie Hospital NHS Trust*
*Wilmslow Road*
*Manchester M20 9BX*

